# Haematuria

**Published:** 1897-12

**Authors:** 


### Haematuria.

EASTERLY, TEXAS, November 5, 1897.

*Editors Texas Medical Journal.*

The enclosed able paper is from the pen of Dr. Ben H. Brodnax, of Brodnax, Louisiana, and is in reply to an article of Dr. McLaughlin on "Hæmaturia," which appeared in the *Texas Medical News*, page 345. It will be seen from this paper that the first case of hæmaturia did *not* occur in 1866, as stated in the *Texas Medical News*, but was known in 1836, just thirty years before.

As to the treatment of Dr. Brodnax, I have given it a fair trial and fully endorse it.

By the way, I admire the remedy suggested by Dr. McLaughlin, which is lager beer. I would not object to the amber colored German fluid, if cold, even if I did not have hæmaturia.

The paper of Dr. Brodnax should have been forwarded sooner, but was mislaid, for which carelessness, on my part, I crave pardon.

W. B. BRIGGS, M. D.
Sec. Brazos Valley Medical Society.

---

BRODNAX, LOUISIANA, October 20, 1897.

W. B. Briggs, M. D. Easterly, Texas.

DEAR DOCTOR—I read with much interest an article in the *Texas Medical News*, page 345. The writer seems to be puzzled on two or three points.

First, the difference of treatment of malarial troubles (particularly the symptom of quinine poisoning, termed hæmaturia) by doctors. I think this is easily accounted for from the fact that text books have for half a century held up the standard, "Quinine for Malaria."

So much has the idea been ground into the student that quinine, and *nothing else*, *would cure malaria*, that they have ceased to think that there might possibly be something else that would take its place.

The *Medical Summary* had a short comment in it some time ago. A dialogue between a doctor and his patient.

"Doctor, I can't take the quinine, it's no use, and it will kill me," said the patient.

"*No it won't!* I tell you—and—and if it does—I can't help it."

Now that is just the position of the pro-quinineites. They won't try anything else, and if the patient is injured, or killed, they can't (or won't) help it. See the August *Brief*, page 1170. (I quote.) "And I unhesitatingly declare that if an applicant for license were to tell me that he had discarded quinine from his armamentarium, that he never used it in malarial disease, no matter how satisfactory and perfect in all other respects his examination might have been, I should refuse to attach my name to his license, even if all the other members of the board desired to pass him." This physician is of your State, and was for several years a member of the examining board of this State. See? Because a doctor refused to use a medicine that he had found, by years of experience, to be dangerous and distressing, the said medical examiner would not allow him to practice in Texas. Has it come to the point that a man must kill his patient, or treat him contrary to his belief, or with nauseating, dangerous medicines in large doses, or quit the State? Texas must be a very rich country, to be able to grow such plants.

The greatest fault I find is that, knowing the distressing effects of quinine on the patient, they *will not cheerfully give a simple, pleasant and sure remedy a fair and impartial trial, strictly according to directions.* There *will be two sides*, as disclosed by the article, until the "new idea" is given a fair chance. Then I think there will be principally one side, with "*pro-quinine*" in the majority.

Secondly, the disease "malarial hæmaturia" was not known in this country *till after the war.* Meeting an old gentleman (ex-justice of the peace, and now one of our police jury) a day or two ago, I asked him if he knew about the date of the first case of swamp yellow fever that he had heard of.

He replied, "Yes, I was overseeing in Tensas Parish in 1855. That was the first case I ever saw, but there were cases in the parish before that. My brother was one of the after cases, in 1856-57. It was brought on him by quinine, and after it was checked they gave the quinine to keep off the next chill; but it came, and the blood with it. I stopped the doctor, then and there, and put him, *myself*, on strychnia and Fowler's solution, with turpentine infusion, and he has had no return of either." So says the judge, a man of fine intellect, too.

"My brother-in-law moved from Mississippi to Carroll Parish, this State, in 1858, at the age of 23. As is customary with new comers, they got to questioning the doctor as to the prevailing diseases of the section. The doctor told me that there were occasional cases of swamp yellow fever in the bottoms, but that they were very uncommon in the hills."

This is the report of the gentleman as to the condition in 1858. There had been, prior to that date, occasional cases in the bottoms. See? 1858, a venerable farmer of our ward, on being asked the status prior to the war, says, "Yes, we had the swamp fever, with hemorrhages from the bladder, some twenty odd years before 1861. It was more common in the swamps than on the uplands. They took quinine, which was a new medicine then, and nearly every case had to quit it, because it brought back the bleeding."

A refugee (during the war) from New Orleans to my native city in Georgia, in describing to me the country of South Georgia, remarked, "Yes, swamp yellow fever (hæmaturia) is not so very uncommon in the swamps back of the city somemiles, but if you don't take quinine you need not be afraid of it." This was in 1861, in Augusta, Georgia, showing that the trouble existed prior to that in South Georgia. So much for the 1866 date of the first case.

Somewhere stowed in my bound copies of medical journals of twenty years ago, I have an article (reprint) sent me by the editor of the *Medical Summary*, giving the history of the socalled "hæmaturia" malarial form, and differentiating it from the Egyptian and West African forms of fever. The date of the reprint is, I think, 1848, but I won't be sure. The pamphlet consists of some six or eight pages, and is a description of a case that occurred on the Maryland shore. I will hunt up the article and send it to you.

Now as to some minor points in the article. Peruvian bark was the article given when I was a boy. It was ground fine like sawdust, steeped in water, and a teaspoonfull of the wet sawdust taken as a dose. The dose then (1840 to 1850) of quinine was one-half to one grain. How much quinine the teaspoonfull of the ground bark contained I cannot say.

"Gunn's Domestic Medicine," edition of 1836, says, "when the fever goes off and you commence sweating take two grains of quinine mixed with a teaspoonfull of epsom salts in water.

Two grains mark you, after fever is subsided; *not 20 to 60 grains after fever comes on.* Rezin Thompson in his "advice" (edition of 1860) advises quinine in dose of 20 grains every three or four hours till "ringing in the ears occurs."

The writer on page 347 refers to the neglected condition of plantations, etc., during and after the war. "I think this has little to do with the disease (malaria). Impure water is the *main* cause. In fact I think 95 out of 100 cases is from impure water, I have known cases of an entire family escaping chills by boiling the drinking water, while of ten other families on same place, who did not take this precaution, scarcely a single one but what was troubled more or less. At same time, as to negroes and whites working in the sun, I have noted that there is little difference as to susceptibility. In the matter of whether quinine does produce (swamp fever) hæmaturia, I could give you quite a number of instances of its *direct action in that* line. A committee appointed by the Tri-State Medical Association to invesgate the charges against quinine last year, reported 76 cases of hæmaturia and in only one case had quinine *not been taken prior* to the hemorrhage; 75 had taken it out of 76. Out of 200 cases thus, there would be about two and a half who *had not taken it.* In my experience of 25 years, I find that *every case* had taken quinine (and not in *2-grain doses* either).

There are those who, run down by chronic chills, poor blood, thinned vessel walls, and general debility; would from very slight cause have the hemorrhage or hæmaglobinuria. *But*, (*and right here stick a pin*) it is not this kind of cases who usually have hæmorrhage from quinine; simply because it has been stuffed down them till it has *no more effect than so much flour*, It is the average healthy man, or woman, who after some days or weeks of the malaria and irritable condition of the system, has a chill, the *quinine given to keep off its recurrence*, shows up, about the time the third dose is taken, and about the moment the chill should *come on in a stream of blood* spouting from the urethra.

I cannot but think that there is some property in quinine sulph that has a poisonous influence on the general system. Its use in 2 to 5 grain doses for several days produces a condition when increased dose must be taken to produce effect, a sort of notion, that the system gets used to it.

But as to the production of hemorrhage from kidneys, bowels, lungs, and on the brain, I am fully satisfied that it is from overstimulation, if an already overloaded and weakened set of blood-vessel that rupture takes place in the parts named.

Also "that if I did not get better results, without any danger, and with a medicine pleasant to take, and with no unpleasant sequela, I would not go back to quinine, but would use strychinia-arsenic and warm turpentine. The acetanelide treatment, whether it alone is used, or "zomatyne" "ammonal," or "antikamnia," which are about 80 per cent acetanelide, 15 per cent some form of soda, 5 per cent caffein or ammo. bi-carb, is the most rational, safe, sure and pleasant mode. It has these qualities, as compared with quinine, and its devilish sequela, I cannot see why it should not be substituted for the quinine way of treatment.

